# Targeted lipidomics reveals a novel role for glucosylceramides in glucose response

**DOI:** 10.1016/j.jlr.2023.100394

**Published:** 2023-05-26

**Authors:** Mark A. Xatse, Andre F.C. Vieira, Chloe Byrne, Carissa Perez Olsen

**Affiliations:** Department of Chemistry and Biochemistry, Worcester Polytechnic Institute, Worcester, MA, USA

**Keywords:** sphingolipids, lipid metabolism pathways, mass spectrometry, monomethyl branched chain fatty acids, phospholipids, elongase, elevated glucose conditions, RNAi, membrane composition, *Caenorhabditis elegans*

## Abstract

The addition of excess glucose to the diet drives a coordinated response of lipid metabolism pathways to tune the membrane composition to the altered diet. Here, we have employed targeted lipidomic approaches to quantify the specific changes in the phospholipid and sphingolipid populations that occur in elevated glucose conditions. The lipids within wild-type *Caenorhabditis elegans* are strikingly stable with no significant changes identified in our global mass spectrometry–based analysis. Previous work has identified ELO-5, an elongase that is critical for the synthesis of monomethyl branched-chain fatty acids (mmBCFAs), as essential for surviving elevated glucose conditions. Therefore, we performed targeted lipidomics on *elo-5* RNAi-fed animals and identified several significant changes in these animals in lipid species that contain mmBCFAs as well as in species that do not contain mmBCFAs. Of particular note, we identified a specific glucosylceramide (GlcCer 17:1;O2/22:0;O) that is also significantly upregulated with glucose in wild-type animals. Furthermore, compromising the production of the glucosylceramide pool with *elo-3* or *cgt-3* RNAi leads to premature death in glucose-fed animals. Taken together, our lipid analysis has expanded the mechanistic understanding of metabolic rewiring with glucose feeding and has identified a new role for the GlcCer 17:1;O2/22:0;O.

Cell membranes are highly organized and dynamic structures made up of hundreds of unique lipids (1, 2). Perturbations including temperature changes and dietary alterations can alter membrane composition and require regulation of metabolic pathways to adjust the makeup of the membrane and optimize its function in the new conditions (3, 4, 5). Of note, elevated concentrations of glucose in the diet of nematodes require an adaptive response coordinated by the membrane sensor, PAQR-2, which is homologous to the mammalian adiponectin receptor. In response to glucose stress and the decreased fluidity associated with high glucose, PAQR-2 activates the desaturase FAT-7, which increases the production of unsaturated fatty acids to restore the fluidity of the membrane (6, 7, 8). Recently, a novel role for the elongase, ELO-5, as part of the response of PAQR-2 to glucose was found using stable isotope labeling and mass spectrometry approaches. Specifically, ELO-5 is required to produce monomethyl branched chain fatty acids (mmBCFAs), and RNAi of *elo-5* results in a sensitivity to glucose diets (9). Although the elevated FAT-7 activity results in the production of unsaturated fatty acids to restore membrane fluidity, the impact of ELO-5 is less clear and does not appear to directly impact fluidity (6, 9).

Distinct from saturated and unsaturated fatty acids, mmBCFAs have a saturated hydrocarbon chain and a methyl group located in the penultimate carbon giving these fatty acids a unique shape (10). In *C. elegans,* the major laboratory diet of *E. coli* (OP50) does not contain mmBCFAs and thus they are entirely synthesized de novo via fatty acid synthase, acyl-CoA synthase, and two fatty acid elongase enzymes, ELO-5 and ELO-6 (11). The mmBCFAs are incorporated into phospholipids (12), and in *C. elegans,* they also serve as precursors for the sphingoid base required to produce sphingolipids (Sphs) (13, 14, 15). mmBCFAs production is essential for larval growth and development, and the roles of these fatty acids in adults have recently started to emerge (16, 17). Recently, it was established that mmBCFAs-derived glucosylceramides (GlcCers) are critical to regulate intestinal apical membrane polarity to allow TORC1 activity in response to nutrient signals in the intestine (18, 19).

GlcCers are considered Sphs and are intermediates of Sph metabolism. Sphs are a highly diverse group of lipid species that contain a sphingoid backbone made up of a linear hydrocarbon chain attached to an amino and hydroxyl group (20). The sphingoid base is amide-linked to long-chain fatty acids usually containing 0 or 1 double bond to form ceramides. Ceramides can further be derivatized by the addition of a head group to form more complex Sphs such as sphingomyelin, GlcCer, and other complex glycosphingolipids (GSLs) with higher numbers of sugar residues (21, 22). Ceramides not only are precursors for complex Sphs but also act as a metabolite with roles in stress response, autophagy, apoptosis, and signaling (23, 24, 25). These processes are often mediated by specific chain length ceramides that are expressed in distinct subcellular compartments that perform unique functions (26, 27). For instance, in *C. elegans*, mmBCFA-derived ceramides are produced by three different ceramide synthases: HYL-1, HYL-2, and LAGR-1. HYL-1 is required to produce ceramides with very long *N*-linked fatty acid (C24 to C26), HYL-2 is required for ceramides with long *N*-linked fatty acids (C20-C22), and LAGR-1 has no major effect on ceramide production (14, 28).

GlcCers are the precursors to more complex GSLs and are formed by the addition of a UDP-glucose to an existing ceramide molecule by ceramide glucosyltransferase (CGT) (29). GSLs impact the structure, localization, and activity of raft associated proteins and, in doing so, influence cellular activities including protein-protein interaction and signaling events (30, 31). In fact, recent studies in *C. elegans* suggest that GSL interacts with cholesterol to form lipid rafts that regulate clathrin localization and signaling processes involved in stress response (32). In *C. elegans*, the production of GlcCer is dependent on ELO-3, a recently characterized elongase that produces the very-long-chain saturated fatty acids included in GlcCer. In addition to supplying precursors for GSLs that have been implicated in stress responses, GlcCers have been implicated as important metabolites for aging in *C. elegans* (32). Because of the crucial roles GSLs play in most organisms, impaired GSL metabolism is linked to many disorders such as cardiovascular diseases, compromised immune response, Gaucher disease, and neurodegeneration (33, 34, 35, 36). However, the complexity of these lipid populations has limited the knowledge of how each lipid species impacts cellular function.

*C. elegans* has become a useful model for the study of lipid metabolism using mass spectrometry and genetic approaches (37, 38, 39). In addition, the small size of *C. elegans* allows for affordable stable isotope labeling to monitor lipid dynamics. Our laboratory has developed ^15^N- and ^13^C- stable isotope feeding strategies in *C. elegans* combined with mass spectrometry to monitor the dynamics of intact phospholipids and fatty acids, respectively (9, 11, 40). Using a combination of approaches, it has been established that mmBCFAs are essential to the nematode’s survival with glucose stress, but it is not known whether this is through a derived lipid, a structural impact on the membrane, or an unknown alternate mechanism (9). Here, we use advanced mass spectrometry to profile the changes in the lipids after glucose exposure, which allows a more thorough understanding of how glucose impacts the phospholipids of the membrane with a particular focus on the Sphs including GlcCer. These lipidomics profiles reveal changes in the ceramide and GlcCer populations following glucose exposure that have been corroborated with glucose survival assays.

## Materials and methods

### Strains and RNAi treatment

All experiments were conducted using wild-type N2 nematodes obtained from the *C. elegans* Genetics Center. For RNAi experiments, RNAi bacteria clones from the Ahringer library [L4440 (empty vector), *elo-5, elo-3,* and *cgt-3*] were grown on NGM + Carbenicillin + IPTG plates (NGM+CI) (41).

### Nematode growth and elevated glucose feeding Protocols

To synchronize the nematodes, gravid adults were exposed to dilute bleach, and the washed eggs were left rotating overnight at 20°C in M9 solution. Approximately 5,000–6,000 synchronized L1 animals were grown at a density of 2,000 worms per 10 cm RNAi treatment plate for 48 h. At L4, nematodes were transferred to NGM+CI plates with (referred to as +gluc plates) or without glucose for an additional 18 h.

The glucose stress was carried out as previously described (9). Briefly, the glucose plates were made to a final concentration of 100 mM glucose by adding a filtered glucose solution to cooled autoclaved NGM+CI medium. The glucose plates were seeded with RNAi bacteria at least 4 days before plating the worms. RNAi bacteria along with the control RNAi were seeded on plates at a density of 0.15 g per 10 cm NGM+CI plate.

### Extraction and Detection of phospholipids by LC-MS/MS

Total lipids were extracted from frozen nematodes via chloroform/methanol (2:1 v/v) solvent system as previously described (40, 42). A 1,2-diundecanoyl-sn-glyerco-3-phosphocholine standard was added for relative quantification later as described previously (Avanti Polar Lipids). Briefly, total lipid extracts were dried under nitrogen and then dissolved in 200 μl of acetonitrile/2-propanol/water (65:30:5 v/v/v) dilution buffer. Next, 10 μl of resuspended lipids were injected onto the LC-MS/MS system for the negative ion scanning mode analysis. Lipid samples were separated using an HPLC system (Dionex UHPLC UltiMate 3000) equipped with a C_18_ Hypersil Gold 2.1 × 50 mm, 1.9 μm column (25002-052130; Thermo Scientific) equipped with a 2.1 mm ID, 5 μm Drop-In guard cartridge (25005-012101; Thermo Scientific). The column was connected to a Dionex UltiMate 3000 RS quaternary pump, a Dionex UltiMate 3000 RS autosampler, and a Q Exactive Orbitrap mass spectrometer from Thermo Scientific coupled with a heated electrospray ionization source.

The HPLC phospholipid separation was carried out with mobile phases A and B consisting of 60:40 water (H_2_O):acetonitrile plus 10 mM ammonium formate (NH_4_COOH) and 0.1% formic acid and 90:10 isopropyl alcohol (IPA):acetonitrile with 10 mM ammonium formate (NH_4_COOH) and 0.1% formic acid, respectively. The gradient method began with 32% B over 0–1.5 min; 32%–45% B from 1.5 to 4 min; 45%–52% B from 4 to 5 min; 52%–58% B from 5 to 8 min; 58%–66% B from 8 to 11 min; 66%–70% B from 11 to 14 min; 70%–75% B from 14 to 18 min; 75%–97% B from 18 to 21 min; 97% B up to 25 min; 97%–32% B from 25 to 26 min; 32% B is maintained until 30 min for column equilibration.

The following parameters were used for the HPLC and MS conditions: column oven temperature was maintained at 50°C and autosampler was set 10°C with mobile phase flow rate at 300 μl/min and MS scan range between *m/z* 300 and 1,200. The capillary temperature was set at 325°C, the sheath gas flow rate was set at 45 units, the auxiliary gas flow was set at 10 units, the source voltage was 3.2 kV, and the AGC target was 10^6^. The acquisition was carried out with full-scan data-dependent MS2 (ddMS2) mode. For MS1 profiling, scans were run at a resolution of 70k. MS2 analyses were performed using six scan events, where the top five ions were chosen from an initial MS1 scan. For fragmentation, a normalized collision energy of 35 was used. MS1 spectra were collected in profile mode, whereas MS2 spectra were collected in centroid mode.

### Extraction and Detection of sphingolipids by LC-MS/MS

Sph extractions were conducted based on previous studies (13, 20, 43). Briefly, total lipids were extracted from nematodes with 2:1 chloroform:methanol for 1.5 h. Ceramide/sphingoid internal standard mixture II (Avanti Polar Lipids) was added prior to extraction. Extracted lipids were dried under nitrogen and 50 μl of 1 M KOH in methanol was added. The mixture was vortexed briefly and incubated at 37°C for 2 h. After incubation, the sample was neutralized with 2–3 μl of glacial acetic acid. After phase separation with 450 μl methanol, 1,000 μl chloroform, and 500 μl of water, the Sph fraction (organic phase) was dried under nitrogen and dissolved in 200 μl of isopropanol/chloroform/methanol (90:5:5, v/v/v).

The Sphs (10 μl injection) were separated and analyzed using the same instrument used for phospholipids with the following modification to the instrument method adapted from a recent study (44). The mobile phase A consisted of water containing 1% formic acid and 10 mM ammonium formate while the mobile phase B consisted of 5:2 (v/v) acetonitrile/isopropanol containing 1% formic acid and 10 mM ammonium formate. The gradient method started at 50% mobile phase B, rising to 100% B over 15 min, held at 100% B for 10 min, and the column was then re-equilibrated with 50% B for 8 min before the next injection. The flow rate was 0.150 ml/min. For MS analysis, Sphs were analyzed in the positive mode with the following parameters: the capillary temperature was set at 275°C, the sheath gas flow rate was set at 45 units, the auxiliary gas flow was set at 10 units, the source voltage was 3.2 kV, and the AGC target was 10^6^. The acquisition was carried out with full-scan data-dependent MS2 (ddMS2) mode. For MS1 profiling, scans were run at a resolution of 70k. MS2 analyses were performed using five scan events, where the top five ions were chosen from an initial MS1 scan. For fragmentation, a normalized collision energy of 50 was used. MS1 spectra were collected in profile mode, whereas MS2 spectra were collected in centroid mode.

### Phospholipid and sphingolipid analysis

Lipid analysis of the LC-MS/MS data was conducted using the software Lipid Data Analyzer (LDA) Version 2.8.1. The LDA utilizes a 3D algorithm relying on the exact mass, predicted isotopic distribution from full scan MS, retention time, and MS/MS spectra for reliable analysis of the lipids (44). During LDA analysis, a 0.1% relative peak cutoff value was applied to the RAW files in order to focus on the major lipid species. LDA mass lists were generated for phospholipids based on a previous study in our laboratory (40). A relative quantification was used to compare between samples; however, the lipids were not normalized, so an absolute quantitation was not done.

LDA mass lists for ceramide and GlcCer were created by combining the carbon length of the sphingoid backbone in *C. elegans* (C17iso SPB) (15, 43) with *N*-linked fatty acid tail ranging from a chain length of 16–28 with degrees of saturation of 0 and 1, which is common for Sphs (21). MS2 scans were manually verified on Xcalibur version 4.1.31.9 for the sphingoid base fragment specific for *C. elegans* of ***250.25***.

Statistical analysis for all the studies were performed using GraphPad Prism 9.4.1 software. Multiple *t*-test (unpaired) with corrected by false discovery rate, with an adjusted p- value (q) at 5% was used to compare two conditions in the lipidomics data.

### Survival analysis

Lifespan was performed with 50 synchronized L4 animals on NGM+CI plates with or without glucose for each replicate. Worms were transferred every day onto fresh plates, and the number of dead animals was confirmed by prodding each animal and recorded daily. For lifespan data, statistical analysis was performed using a log-rank (Cox-Mantel) test.

### Sphingolipid supplementation

Sph were extracted from adult WT animals (grown for 66 h post L1) as described above. Ethanol was added to dried Sphs at density of ∼50,000 worms per 5 ml of ethanol. The solution was filtered with a 22 μm syringe and stored in −20°C. For lifespan analysis, 50 μl of Sph extract or ethanol was pipetted per 3 cm NGM +CI plates seeded with RNAi bacteria. For large-scale lipidomics analysis 500 μl of Sph extract or ethanol was pipetted per 10 cm NGM +CI plates.

## Results

### Phospholipid populations with glucose exposure

**Fig. 1:**
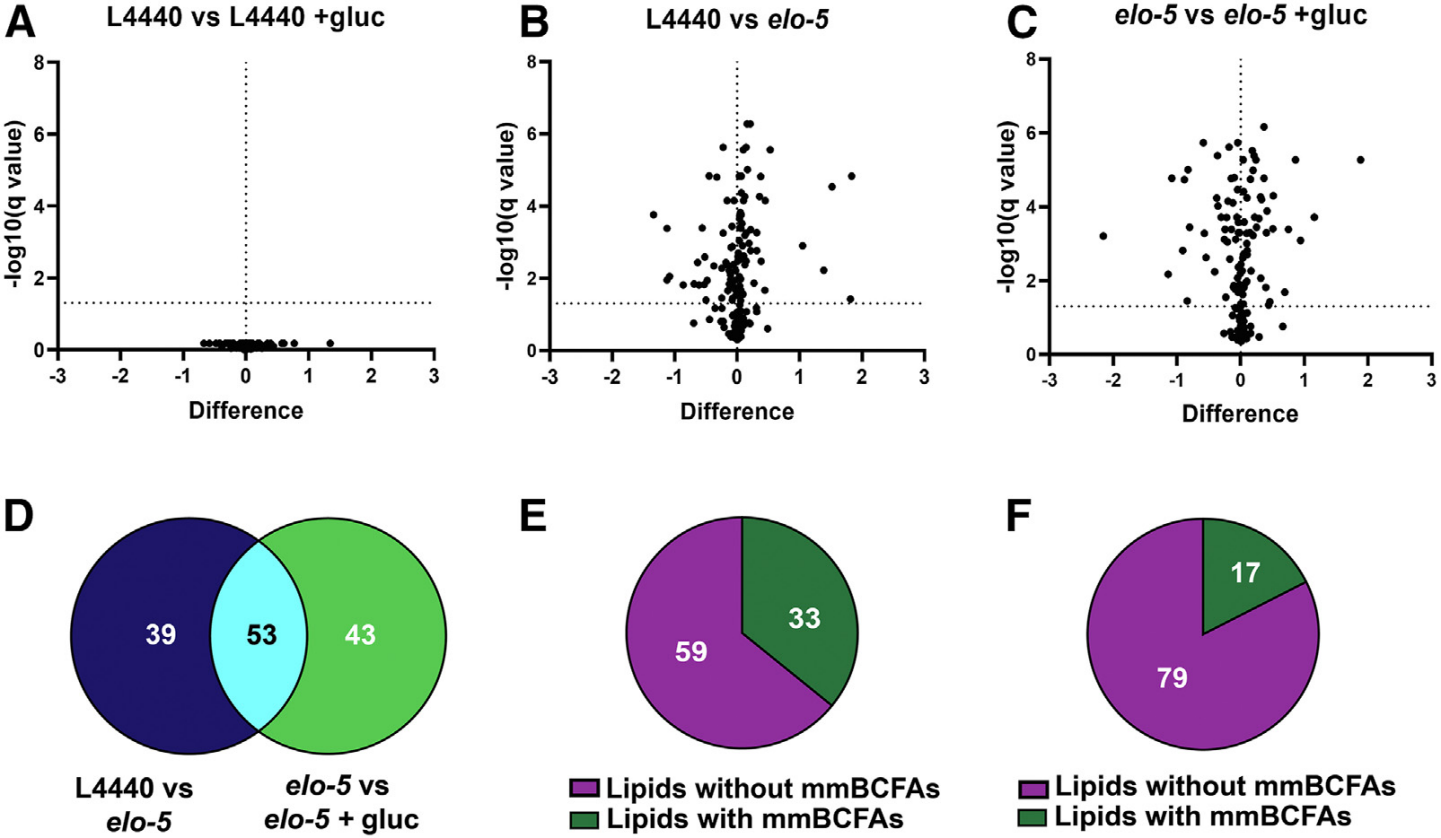
Global phospholipidomics were determined after 100 mM glucose exposure. WT animals at L1 stage were fed RNAi (L4440 or *elo-5*) on NGM+CI plates for 48 h followed by 18 h feeding on NGM+CI plates with or without 100 mM glucose. A total of 159 phospholipids (PL) were characterized, namely, phosphatidylcholines, PC (50); phosphatidylethanolamines, PE (56); lysophosphatidylcholines, LPC (14); lysophosphatidylethanolamine, LPE (20); plasmanyl ethanolamine, PE P- (11); and plasmenyl ethanolamine, PE P- (8). The relative abundance (normalized to total lipids) was quantified, and the volcano plot shows the fold change in the relative abundance of distinct phospholipid species. Each dot above the dashed lines in the volcano plot are significantly changed with the upper right and upper left panels showing increased and decreased lipid species, respectively. Volcano plot here shows (A) no significant difference between L4440 worms fed with and without glucose, (B) significant changes in the PL composition of L4440 compared with *elo-5*-fed worms, and (C) significant changes between *elo-5*-treated worms with and without glucose. D: Venn diagram shows phospholipid species that are common and distinct between species that are significantly changed in L4440 versus *elo-5* compared with *elo-5* versus *elo-5 +gluc.* The results indicate that there are independent changes from each group with 53 phospholipids common between both groups. E and F: show the distribution of phospholipid species that are significantly changed in (B) and (C), respectively, that have a monomethyl branched-chain fatty acid (mmBCFAs) in at least one of the phospholipid tails as determined by MS2 fragments in the Lipid Data Analyzer software. Data were generated from at least four independent biological replicates; statistical significance was calculated by multiple *t*-test corrected by false discovery rate, with an adjusted p-value (q) at 5%.

Although it is clear that fatty acid metabolism is perturbed with excess dietary glucose, the specific changes in the phospholipids of the animals have not been defined. Here, we profiled the phospholipids of control RNAi animals in the presence of 100 mM glucose. Populations of nematodes (L4 stage) were grown with (+gluc) and without 100 mM glucose for 18 h and membrane lipids [i.e., phosphatidylcholine (PC), phosphatidylethanolamine (PE), lysophosphatidylcholine (LPC), lysophosphatidylethanolamine (LPE), plasmanyl ethanolamine (PE O-) and plasmenyl ethanolamine (PE P-)] were analyzed using a Q Exactive mass spectrometer (Thermo Scientific, Waltham, MA). The relative abundance of the lipids was determined using Lipid Data Analyzer software and the fold change of distinct lipids under glucose stress compared. The results showed no significant differences in any of the phospholipids in the target list after 18 h of glucose exposure (Fig. 1A; complete list found as supplemental Table S1). The lack of significant changes in wild-type nematodes fed glucose is not surprising as it has been established that N2 animals can adapt to high-glucose diets (6, 9). In addition, these populations build most of the membrane, and the glucose exposure is relatively short, minimizing the impact of metabolic changes on the entire population due to dilution effects.

We next examined the impact on the phospholipid population when the glucose response is compromised by using an *elo-5* RNAi feeding approach. Here, *elo-5* RNAi was initiated at L1 stage for 48 h, and then the animals were transferred to plates with and without glucose for 18 h. First, to establish a baseline comparison, *elo-5* RNAi and control RNAi-fed animals were compared and the results reveal that ∼58% of the lipids (92/159) were significantly changed with *elo-5* RNAi (Fig. 1B). Next, *elo-5* RNAi-treated animals were subjected to 18 h of glucose exposure. This glucose treatment led to significant changes (∼60% of lipids measured) in the phospholipid composition indicating that the ability to maintain membrane composition under glucose stress is compromised with the loss of ELO-5 (Fig. 1C). Because there are a significant number of alterations that occur with *elo-5* RNAi feeding with and without glucose, we compared the species with significant changes and found that only 53 phospholipid species were altered in both conditions (Fig. 1D). There are 43 phospholipid species that are significantly impacted when *elo-5* RNAi is combined with glucose treatment implicating these species in the glucose response. Because ELO-5 is only involved in the production of C15iso and C17iso fatty acids, the impact on this large percentage of membrane lipids implicates ELO-5 or a product of this enzyme in regulating phospholipid metabolism on a larger scale.

To interrogate this further, the MS2 scans of the significantly changed species were used to determine if the lipid contains an mmBCFA in at least one of its tails. In *elo-5* RNAi-fed populations, only 35.8% of the altered species contain a mmBCFA when compared with L4440 controls (Fig. 1E). In *elo-5* RNAi-fed animals treated with glucose, only 17.7% of the altered species contain a mmBCFA compared with *elo-5* RNAi without glucose (Fig. 1F). Since most of the changed species do not directly contain an mmBCFA, the role of ELO-5 in glucose response is more wide reaching than just the production of these fatty acid species for inclusion in phospholipids.

### Analysis of phospholipid classes and the associated fatty acid tails

**Fig. 2:**
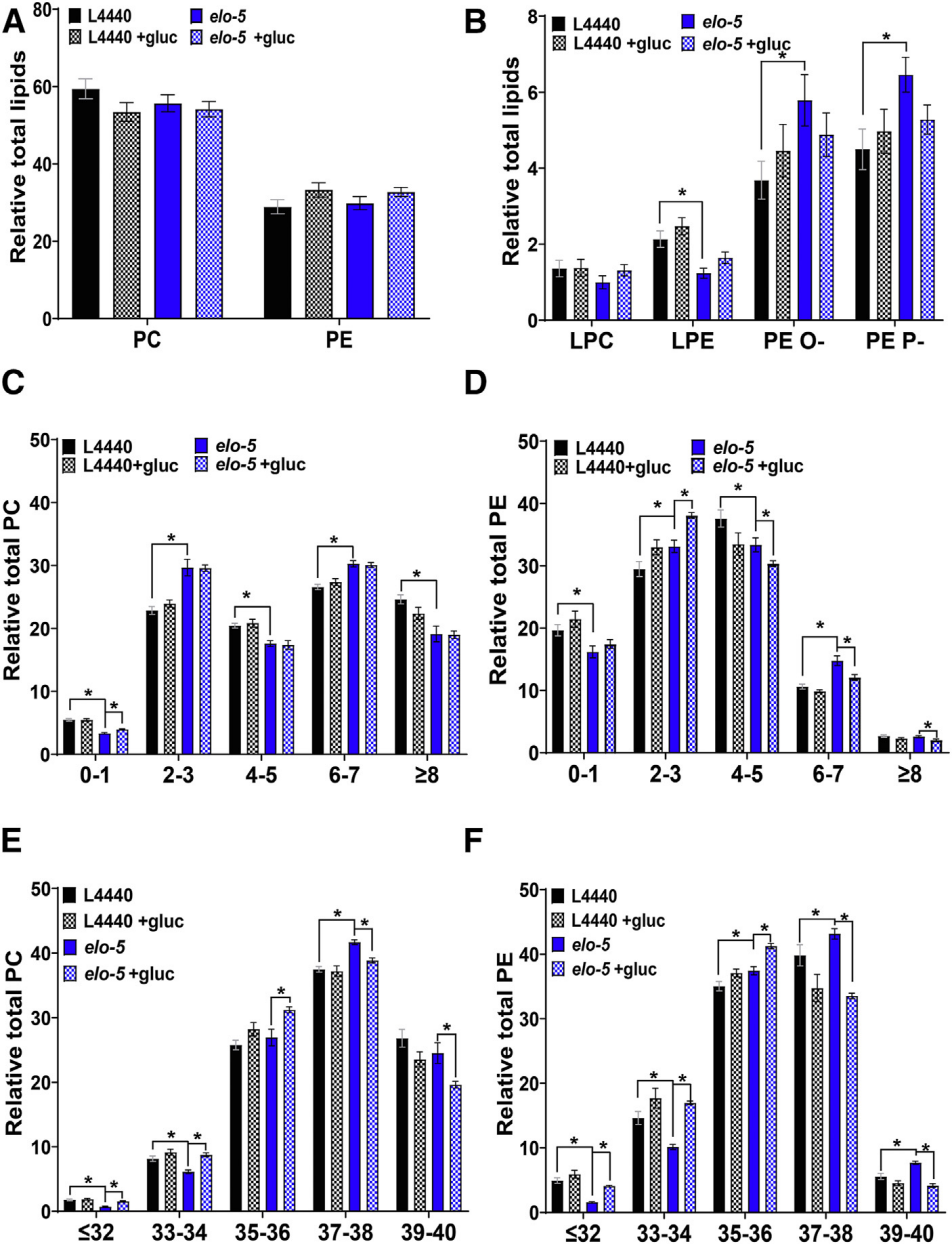
Glucose feeding impacts fatty acid saturation and length in phospholipids. A: The relative distribution of the major PL class, PC and PE, for L4440 (black), L4440 +gluc (black and checkered), *elo-5* (blue), and *elo-5* +gluc (blue and checkered) was generated by targeted lipidomic analysis. B: The distribution of the less abundant lipid classes, LPC, LPE, PE O- and PE P-, was generated for the same populations of animals. The results show a significant decrease in the LPE population and a significant increase in the plasmalogen (PE O- and PE P-) species in L4440 compared with *elo-5* RNAi. Glucose stress did not significantly impact the distribution of each lipid class. C: PC phospholipids were binned by the total number of double bonds associated with their fatty acids for each treatment group. The result shows a significant decrease in PL with a total of 0–1, 4–5, and 8 or more double bonds and an increase in lipids with 2–3 and 6–7 double bonds in L4440 compared with *elo-5* RNAi. Glucose stress did not greatly alter the distribution of double bonds apart from a significant increase in PC species with 0–1 double bonds in *elo-5* glucose-stressed animals. D: Similarly, the double bond distribution of PE lipids was quantified. The results showed a significant increase in PE species with 6–7 and 2–3 double bonds and a decrease in lipids with 0–1 and 4–5 double bonds in L4440 compared with *elo-5*. Glucose stress did not alter the PE double bond distribution in control animals but led to a significant increase in PE species with 2–3 double bonds and a significant decrease in species with 4 or more double bonds in *elo-5* animals stressed with glucose. E: PC lipids were binned according to the total number of carbons in the fatty acid tails. The results indicate that the loss of *elo-5* significantly increased lipids with a total fatty acid tail of 37–38 and a significant decrease in lipids with a relatively shorter chain (less than 36 carbon). Glucose did not impact the chain length of control animals but led to significant increase in shorter-chain lipids (less than 36) and a decrease in longer chains. (36 carbon or more). F: Similarly, PE lipids binned according to chain length show a significant decrease in lipids with shorter chain length (less than 36 carbons) and a significant increase in lipids with longer carbon chains (36 or more) in *elo-5* RNAi worms compared with controls (L4440). Contrastingly, the presence of glucose showed a significant increase in lipids with relatively shorter carbon chains (36 carbons or less) and a significant decrease in lipids with longer carbon chains (more than 36 carbons) in *elo-5* worms stressed with 100 mM glucose while controls remained unchanged. Data were generated from at least four independent biological replicates. Statistical significance was calculated by multiple *t*-test corrected by false discovery rate, with an adjusted p-value (q) at 5%.

To further investigate the impact of ELO-5 across lipid metabolism pathways, the relative distribution of each phospholipid class was determined. In comparing control and *elo-5* RNAi fed animals, there was not a significant difference between the PC and PE headgroups between L4440 and *elo-5* RNAi (Fig. 2A). Similarly, there was no change in PC and PE distribution with 100 mM glucose feeding of either control or *elo-5* RNAi (Fig. 2A, see supplemental Table S2 for complete list). We next probed lipid populations that are less abundant but play critical roles in signaling and stress response, specifically the lysophospholipids and the plasmalogens (42, 45). The abundance of LPE is significantly lower in *elo-5* RNAi when compared with controls (1.24 ± 0.13% vs. 2.13 ± 0.22%). The reduction in the LPC and LPE population in the *elo-5* RNAi-treated animals is largely from the depletion of lysophospholipids containing an mmBCFA tail, suggesting that the pool of precursors for mmBCFA-containing fatty acids is severely limited (supplemental Table S1 for full list). The abundance of the LPC and LPE was not altered by glucose exposure in control RNAi and *elo-5* RNAi populations, suggesting that this reduction in LPE is due to a lack of ELO-5 activity in building the membrane lipids but not in the response to short-term glucose stress (Fig. 2B). In analyzing the plasmalogens, we observed an increase in both PE O- and PE P- abundances in *elo-5* RNAi-treated animals from 3.68 ± 0.50% to 5.79 ± 0.67% and from 4.50 ± 0.53% to 6.46 ± 0.46%, respectively (Fig. 2B). Despite the elevated PE O- and PE P- levels in untreated *elo-5* RNAi animals, again the glucose exposure does not lead to an additional increase in the *elo-5* RNAi animals suggesting that these animals have a compromised lipid pool and that plasmalogens are not an induced response to glucose.

The types of fatty acids in each pool have a dramatic impact on lipid metabolism and membrane function (1, 46). We analyzed the overall degree of saturation within the PC and PE classes as these glycerophospholipids comprise ∼88% of the total phospholipid population. First, the PC species were sorted in bins by the number of total double bonds within the animal to define the overall level of saturation in the phosphatidylcholine lipids in the control RNAi and the *elo-5* RNAi-fed animals without glucose (Fig. 2C). In animals fed *elo-*5 RNAi, there are alterations in the distribution of unsaturated lipids with a significant increase in PC lipids with 2–3 and 6–7 double bonds and a significant decrease in PC lipids with 4–5 and ≥8 double bonds. Importantly, there is a significant decrease in the 0–1 double bond bin between control RNAi and *elo-5* RNAi that includes mmBCFAs (Fig. 2C). The addition of glucose did not alter the level of unsaturation of the majority of PC lipids in control and *elo-5* RNAi worms except for a small but statistically significant increase in the 0–1 double bond bin (Fig. 2C). In analyzing the level of unsaturation in the PE population, there were fewer alterations between control RNAi and *elo-5* RNAi than in the PC; however, we did observe a significant decrease in the 0–1 double bond bin, consistent with mmBCFA loss, and a decrease in 4–5 double bonds. In addition, there was a significant increase in the level of PE lipids with 2–3 double bonds and 6–7 double bonds (Fig. 2D). The addition of glucose did not impact the level of saturation of control animals, but interestingly the addition of glucose significantly increased the levels of lipids with 2–3 double bonds and significantly decreased lipids with ≥4 double bonds in *elo-5* RNAi-treated worms (Fig. 2D). Overall, the phospholipidomic analysis indicates that most significant differences in *elo-5* RNAi-treated animals is due to loss of the enzyme, but there is a significant role for *elo-5* in the alterations of PE lipids in animals treated with glucose.

The biophysical properties of phospholipids are also impacted by the chain length of the associated fatty acid tails (47, 48). Therefore, we performed a similar analysis, but binning based on chain length (i.e., ≤32, 33–34, 35–36, etc. carbons in both fatty acid chains). Like the double bond analysis, there are multiple significant changes between control RNAi and *elo-5* RNAi-fed animals in the PC (Fig. 2E) and the PE classes (Fig. 2F), specifically decreases in ≤32 and 33–34 carbons. Interestingly, these decreases are offset in each lipid class differently with an increase in 37–38 for PC and in 35–36, 37–38, and 39–40 for PE, indicating that the regulation is not occurring in the fatty acid pool but in phospholipid metabolism pathways. The addition of glucose shows no impact on the chain length in control animals; however, there are several significant changes in *elo-5*-treated animals fed high-glucose diets. Here, the chain length changes are similar in both PC (Fig. 2E) and PE (Fig. 2F): elevated ≤32, 33–34, and 35–36 bins and decreased 37–38 and 39–40 bins. These changes cannot be explained by the reduced capacity to produce mmBCFA and further support that ELO-5 plays roles in regulating metabolism perhaps through impacting signaling lipids.

### Glycolipid populations respond to glucose diets

**Fig. 3:**
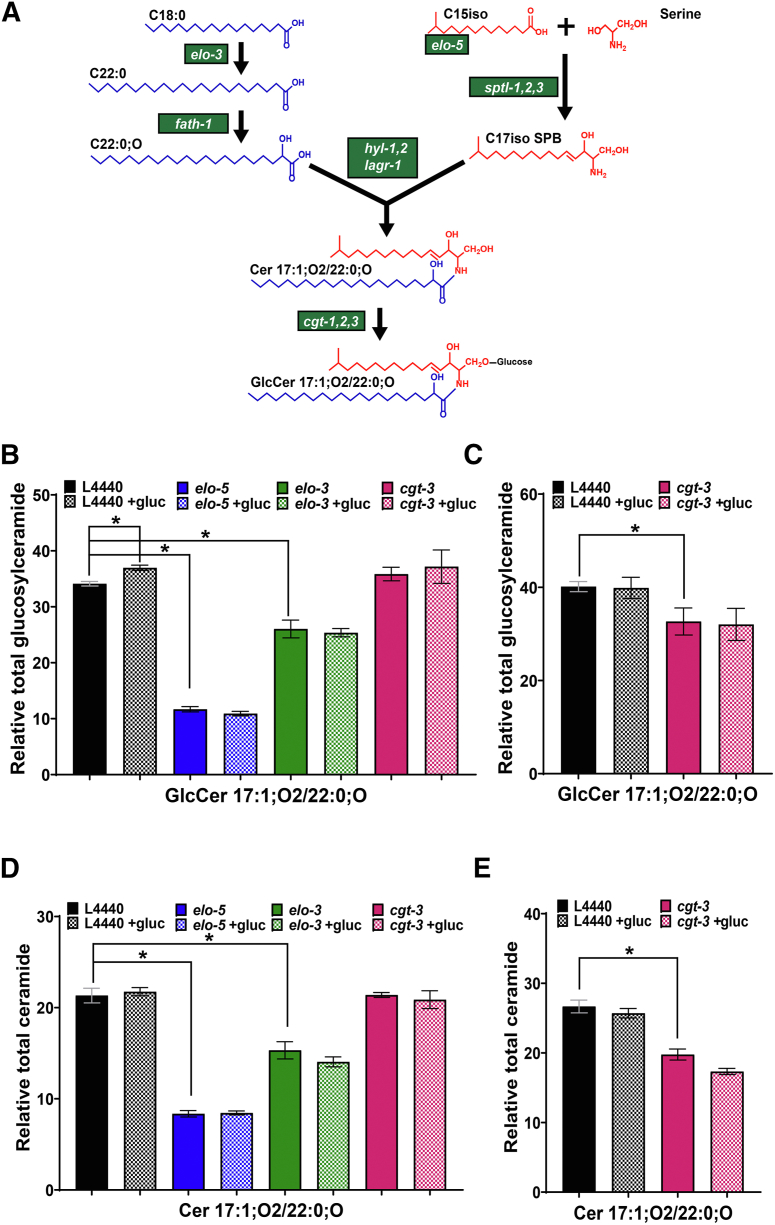
RNAi of enzymes in the sphingolipid pathway in *C. elegans* compromises the level of GlcCer 17:1;O2/22:0;O. A: Glucosylceramides (GlcCer) are synthesized from C15iso and saturated fatty acid (C18:0) through a series of steps catalyzed by various enzymes (highlighted in green) (adapted from Zhu *et al.*, 2013 (19)). B: WT animals at L1 stage were fed RNAi (L4440, *elo-5, elo-3,* and *cgt-3)* on NGM+CI plates for 48 h followed by 18 h feeding on NGM+CI plates with or without 100 mM glucose. Following alkaline hydrolysis of phospholipids, the ceramide and glucosylceramide levels were measured using HPLC-MS/MS. The level of GlcCer 17:1;O2/22:0;O is significantly increased in control animals exposed to 100 mM glucose. *elo-5* and *elo-3* RNAi-fed animals have lower levels of GlcCer 17:1;O2/22:0;O and exposure to glucose does not further increase the levels of this sphingolipid. Surprisingly, *cgt-3* did not alter the levels of GlcCer 17:1;O2/22:0;O. C: Longer *cgt-3* RNAi treatment (3 days after L4 stage) led to a significant decrease in the level of GlcCer 17:1;O2/22:0;O. D: In analyzing the ceramide precursor for GlcCer 17:1;O2/22:0;O- Cer 17:1;O2/22:0;O, *elo-5* and *elo-3* have significantly lower levels compared with control animals, while *cgt-3* levels are not impacted. Glucose stress did not impact the level of Cer 17:1;O2/22:0;O in any of the treatment conditions. E: Longer *cgt-3* RNAi treatment led to a significant decrease in the level of Cer 17:1;O2/22:0;O. Data were generated from at least three to five independent biological replicates. Statistical significance was calculated by multiple *t*-test corrected by false discovery rate, with an adjusted p-value (q) at 5%. For the purposes of clarity, the statistical comparison shown in the graphs are between controls and RNAi backgrounds and the change in abundance of each background upon glucose exposure for GlcCer 17:1;O2/22:0;O and Cer 17:1;O2/22:0;O.

The GlcCer population is of particular interest in *elo-5*-treated animals as the mmBCFA population provides precursors for their synthesis. In particular, GlcCer 17:1;O2/22:0;O is a critical metabolite that is responsible for regulating postembryonic growth and development but has not been implicated in response to glucose (19). The Lipid Maps nomenclature for Sphs is GlcCer XX/YY;O where GlcCer represents the headgroup, XX represents the sphingoid base, YY represents the *N*-linked FA, and O represents the hydroxyl group attached to the *N*-linked FA (49). Because all the GlcCer species examined here contain a d17iso sphingoid backbone, we will report the associated fatty acid chain only for readability. For example, GlcCer 17:1;O2/22:0;O will be referred to as 22:0;O GlcCer. The synthesis of 22:0;O GlcCer requires ELO-5 to produce the mmBCFA backbone and ELO-3 to produce saturated very-long-chain fatty acids, which are both further processed before ultimately being joined to glucose by a CGT to produce 22:0;O GlcCer (Fig. 3A) (19, 32). In order to determine if the 22:0;O GlcCer has a role in glucose response, we first established an assay by HPLC-MS/MS to quantify the abundance of this species based on a method previously developed (13, 43). Although our method does not distinguish between the isomeric hexosylceramides (HexCer), GlcCer, and galactosylceramide, there are no data available currently about the presence of galactosylceramide in *C. elegans*. The HexCer measured in *C. elegans* in previous studies is GlcCer (13, 19, 29, 32) and for the rest of the paper, we assume that the HexCer measured here is GlcCer. Animals treated with *elo-3* RNAi were included as a control as ELO-3 is required to produce 22:0;O GlcCer and indeed the abundance of this lipid was significantly reduced from 34.12 ± 0.40% in control animals to 26.07 ± 1.59% with *elo-3* RNAi validating the methodology (Fig. 3B, supplemental Table S3). Similarly, *elo-5* RNAi led to strongly reduced 22:0;O GlcCer to 11.69 ± 0.47% (Fig. 3B).

*C. elegans* has three CGTs, *cgt-1,2,3*. We focused on the impact of *cgt-3* RNAi because CGT-3 protein was previously shown to have the highest CGT activity compared with *cgt-1* and *cgt-2* (50) and has also been recently shown to be important for stress response (32). Interestingly, there was no significant reduction in 22:0;O GlcCer with *cgt-3* RNAi; however, we observed death in the *cgt-3* RNAi suggesting the RNAi was effective (Fig. 3B). We hypothesized that this lack of reduction may be a result of the relatively short-term RNAi exposure. To test this, we extended the RNAi period to 3 days at which point we observed significant death within the *cgt-3* RNAi-treated animals. In these longer term *cgt-3* RNAi-treated animals, we were able to quantify significantly reduced 22:0;O GlcCer but not to the same extent as with *elo-5* and *elo-3* RNAi, which we hypothesize is a result of the death of the animals with the most reduced levels (Fig. 3C, supplemental Table S4). Because of the power of HPLC-MS/MS, the levels of 22:0;O Cer, the immediate precursor to 22:0;O GlcCer, were also quantified. The same trends were observed in this population with significantly reduced 22:0;O Cer in *elo-5, elo-3*, and long-term *cgt-3* RNAi treatment (Fig. 3D, E).

Once the assays were validated, the abundance of 22:0;O GlcCer was quantified after 100 mM glucose exposure, and there was a significant increase from 34.12 ± 0.40% to 36.96 ± 0.48% in control animals (Fig. 3B). There was no increase in 22:0;O GlcCer in *elo-3, elo-5*, or long-term *cgt-3* RNAi treatment indicating that this elevation with glucose treatment is due to increased synthesis of 22:0;O GlcCer and not reduced consumption or degradation (Fig 3B, C). Interestingly, glucose exposure did not lead to an increase in the production of 22:0;O GlcCer with long-term glucose treatment in control populations implicating the increased production as a short-term impact that is modulated with time. Similarly, there is no change in the 22:0;O Cer pool in controls treated with glucose (Fig. 3D) suggesting that there is an immediate role for GCT in funneling precursors to 22:0;O GlcCer production.

### Ceramide and Glucosylceramide pools change with high-glucose diets

**Fig. 4:**
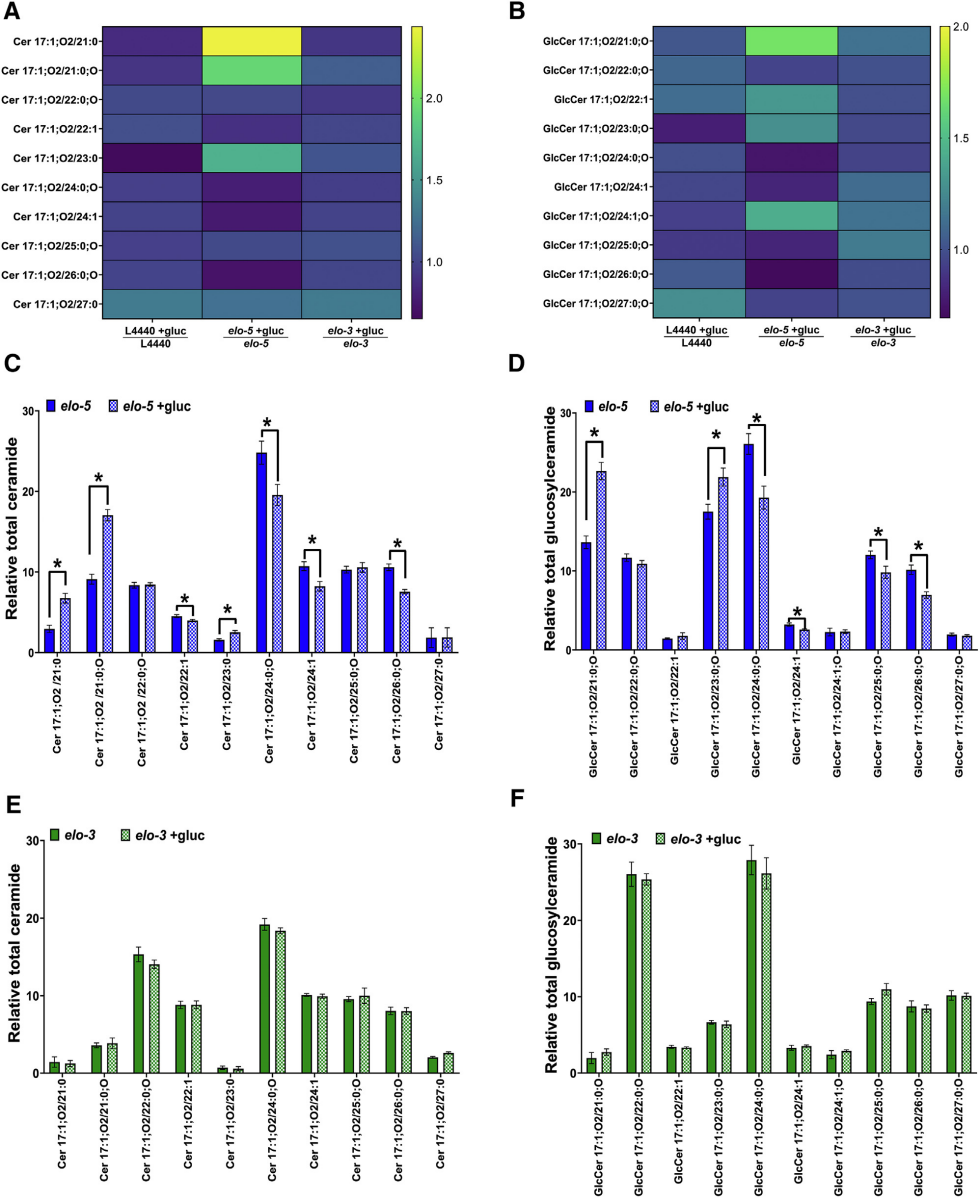
Sphingolipid profile shifts after 100 mM glucose exposure. The other sphingolipid populations in animals treated in the conditions described in Fig 3B were measured. The heatmap shows the fold change after glucose stress (mean of RNAi-treated worm/mean of glucose stress RNAi) for the ceramides that are precursors to GlcCers (A) and all the glucosylceramides measured (B) detected in *C. elegans* under different RNAi backgrounds (see full target list in supplemental Table S3). The results show a unique response for each RNAi-treated worm group under glucose conditions with *elo-5* animals having significant shifts with glucose treatments. Specifically, in *elo-5* animals fed with glucose compared with *elo-5* animals (C) ceramides with odd-chain FAs (21:0 Cer, 21:0;O Cer, 23:0 Cer) were significantly increased while species with even-chain fatty acids (22:1 Cer, 24:0;O Cer, 24:1 Cer, 26:0;O Cer, 26:1) were significantly decreased. D: This trend was consistent in the GlcCer pool with significant increase in shorter and odd-chain GlcCers (21:0;O GlcCer, 23:0;O GlcCer) and significant decrease in even-chain GlcCers (24:0;O GlcCer, 24:1, and GlcCer, 26:0;O). Interestingly, there were no significant changes in the ceramides species shown here (E) and glucosylceramide species (F) in *elo-3* compared with *elo-3* animals stressed with 100 mM glucose. Data were generated from at least three to five independent biological replicates. Statistical significance was calculated by multiple *t*-test corrected by false discovery rate, with an adjusted p-value (q) at 5%.

Although 22:0;O GlcCer has been implicated in other phenotypes of mmBCFA deficiency, there are many other GlcCer and ceramide species within the nematode. We next quantified the effects of glucose exposure on all the major GlcCers or the nematode along with ceramide species that are precursors to those GlcCers (Fig. 4A, B) (see supplemental Table S3 for full list). First, we examined lipid species that changed with glucose exposure in L4440 RNAi animals and found that the majority of ceramides and GlcCers are stable except there was a slight significant decrease in 23:0 Cer and a significant increase in 22:1 GlcCer. Overall, this suggests that the response to glucose in the diet alters Sph metabolism to keep the levels of most individual species relatively stable (Fig 4A, B, supplemental Table S3).

We next interrogated how these lipid populations are impacted without the ability to synthesize new Sph molecules. Because *elo-5* RNAi had the greatest impact of the 22:0;O GlcCer pool, we first compared the Sph profiles in *elo-5* RNAi-fed animals that were also subjected to high-glucose diets. There were significant alterations in ceramides with species that have shorter (≤23 carbons) FA attached to the sphingoid backbone; specifically, 21:0 Cer, 21:0;O Cer, and 23:0 Cer increased significantly by ∼144%, 93%, and 67%, respectively (Fig. 4C). Interestingly, this trend was observed for the odd-chain FA but not the even-chain FA. Conversely, there were decreases in several species that have longer chains (≥24), namely, 24:0;O Cer, 24:1 Cer, 26:0;O, and Cer, 26:1 Cer, which decreased significantly by ∼21%, 23%, 28%, and 28%, respectively (Fig. 4C). In the GlcCer pool, there were significant changes in 6 of 10 species with trends similar to the ceramide pool. Specifically, species with shorter (≥23) and odd FA attached to the sphingoid backbone including 21:0;O GlcCer and 23:0;O GlcCer are significantly increased by ∼69% and 27%, respectively (Fig. 4D). GlcCer species with longer (≤24) and even FA sphingoid backbone, namely, 24:0;O GlcCer, 24:1 GlcCer, and 26:0;O GlcCer, were significantly decreased, by ∼26%, 20%, and 31%, respectively (Fig. 4D). Because ELO-5 is not directly responsible for the synthesis of the long-chain FA associated with these ceramides, the significant perturbation of the ceramide pool further implicated ELO-5 in broader regulation of Sphs following glucose stress.

We next examined the impact of compromising ELO-3, which is directly important in synthesizing the long-chain FA found in the ceramide and GlcCer populations. Interestingly, we found that *elo-3* RNAi does not have any major effect on the Cer and GlcCer pools under glucose stress (Fig. 4E, F), with the only significant change observed being a decrease in 25:0 Cer (supplemental Table S3). The more minimal effect on the Sph pool may be due to compensatory pathways or a milder decrease in enzyme levels by the *elo-3* RNAi clone. Regardless, the impact of glucose stress on ceramide and GlcCer is more severe in *elo-5*-fed RNAi animals compared with control and *elo-3*-fed animals. The specificity of ELO-5 in impacting the overall Sph pool even when ELO-5 is not directly implicated in synthesizing components strongly suggests that ELO-5 is responsible for producing a specific species that orchestrates a shift in the composition of the Sph pool. We predict that this species is 22:0;O GlcCer, which is the most depleted in *elo-5* RNAi-fed animals.

### Sphingolipid synthesis is critical for survival in elevated glucose conditions

**Fig. 5:**
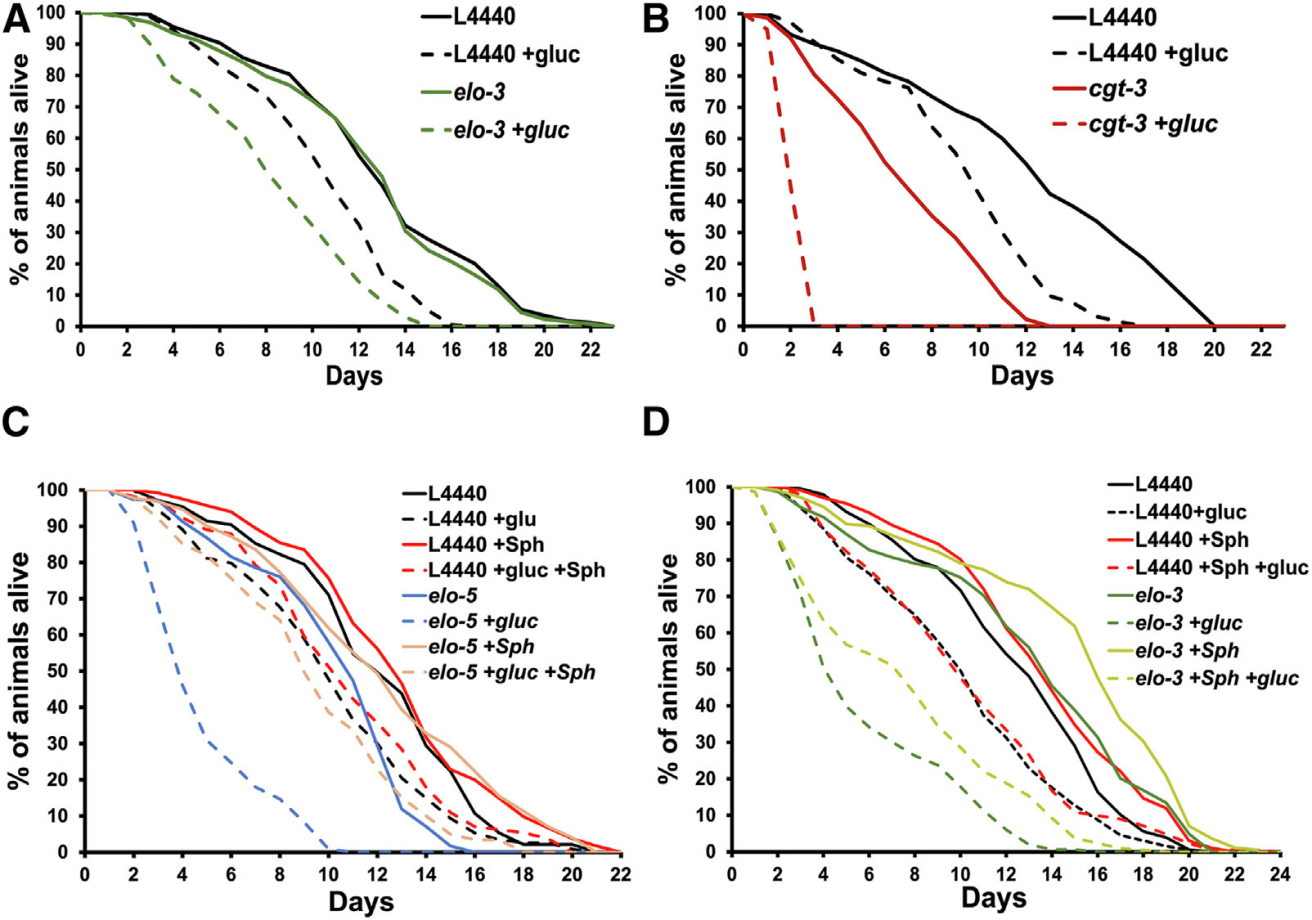
*elo-3* and *cgt-3* RNAi decrease the survival of the nematodes under glucose stress. A: RNAi knockdown of *elo-3* (green) and control (black) in N2 worms was initiated from the L1 stage. After 48 h (L4 stage), the animals were transferred to NGM+CI plates with (dashed line) or without (solid line) 100 mM glucose (+gluc). The loss of ELO-3 significantly shortened the mean (± SD) lifespan of WT animals under glucose stress from 9.6 ± 1.5 days to 7.5 ± 1.6 days. B: *cgt-3* (red) RNAi knockdown animals had a dramatic decrease in lifespan under glucose stress with a maximum lifespan at 3 days. C: To further interrogate whether *elo-5* sensitivity to glucose was through the production GlcCer, *elo-5* RNAi animals were supplemented with sphingolipids (Sph) purified from WT animals (light red) or ethanol control (blue). RNAi was initiated ∼12 h after L1 to prevent the smaller size and shorter lifespan observed when *elo-5* RNAi is initiated at L1 (9). Supplementing *elo-5* RNAi with purified sphingolipid was able to restore the mean lifespan from 5.0 ± 0.5 days to 9.6 ± 0.8 days. D: Similarly, *elo-3* RNAi animals were supplemented with sphingolipid extract. The addition of the supplement partially rescued the survival of *elo-3* animals from mean lifespan of 6.0 ± 1.4 days to 7.6 ± 2.0 days. The survival curves are presented as means of at least three to five independent replicates with 50 worms per condition for each replicate. The statistical analysis of the survival curve was performed by the Log-rank (Mantel-Cox) test.

Because of the altered 22:0;O GlcCer levels observed in the *elo-3* RNAi animals, we examined whether *elo-3* RNAi also caused glucose sensitivity. Synchronized L1 animals were fed control or *elo-3* RNAi, and, after 48 h, L4 stage animals were transferred to RNAi plates with or without 100 mM glucose. The results show that *elo-3* RNAi significantly decreased the median lifespan of WT animals under glucose stress from approximately 11 days to 8 days (Fig. 5A). Because the majority of the Sph pool is stable with *elo-3* RNAi except for 22:0;O GlcCer, this result suggests that this lipid has a specific effect on glucose survival. We cannot distinguish whether the *elo-3* RNAi animals survive better than *elo-5* RNAi animals because of the reduced impact on 22:0;O GlcCer levels or because of the alterations in the other Sph pools that are only seen with *elo-5* RNAi.

Next, to further confirm the need for 22:0;O GlcCer in glucose response, we examined the survival of nematodes under glucose stress in the absence of the CGT. As previously described, we focused on *cgt-3* RNAi based on its established role in stress response. Interestingly, *cgt-3* RNAi dramatically shortened the lifespan of the animals under glucose stress with a maximum lifespan of 3 days (Fig. 5B). This dramatic decrease in lifespan was more severe than the shortened lifespan observed previously in *elo-5* RNAi animals (9). We next wanted to determine if this sensitivity was unique to CGT-3, so we monitored glucose stress survival with cgt-1 RNAi as well. There was no significant effect on the survival of the *cgt-1* RNAi-fed animals under glucose stress (supplemental Fig. S1A). Furthermore, the combination *cgt-3* and *cgt-1* RNAi (1:1 ratio) did not exacerbate the *cgt-3* lifespan decrease (supplemental Fig. S1A). This result suggests a critical role of GlcCer production in the survival of glucose stress.

Because the sensitivity of *cgt-3* RNAi-fed animals strongly suggests a role for GlcCers in adapting to elevated glucose conditions, we next fed animals a combination of *cgt-3* and *elo-5* RNAi and quantified survival with glucose treatment. The combined *cgt-3/elo-5* RNAi treatment did not increase the rate of death in the glucose-treated nematodes suggesting that these genes function in the same pathway in response to glucose (supplemental Fig. S1B). It is important to note that the *cgt-3* RNAi treatment leads to very rapid death with all animals dying within 4 days; therefore, it is possible that additive effects with the *elo-5* RNAi would not be detectable.

Next, to confirm that the *elo-5* RNAi acts through the GlcCer pathway and not through an alternative mmBCFA function, we sought to rescue the *elo-5* RNAi-treated animals with GlcCers. To do so, we purified Sphs that contain the Cers and GlcCers from wild-type nematodes (the composition of the Cers and GlcCers is found as supplemental Table S5 and supplemental Fig. S1C,D). The purified Sphs were fed to *elo-5* RNAi-treated animals in the presence and absence of glucose (Fig. 5C). The addition of WT Sph to RNAi control animals (mean lifespan of 12.5 ± 1.6 days) did not significantly impact the lifespan of control animals (mean lifespan of 12.1 ± 0.5 days). However, the addition of WT Sph to the *elo-5* RNAi animals restores the lifespan of these animals (mean lifespan, 12.1 ± 1.4 days) to wild-type levels (Fig. 5C). The impact of WT Sph on *elo-5* animals' survival in the presence of glucose is even more dramatic with a mean lifespan increased to 9.6 ± 0.8 days from 5.0 ± 0.5 days (Fig. 5C). Taken together, these lifespan curves implicated GlcCer production as the primary critical role for ELO-5 in the nematode particularly in response to glucose. Similarly, we supplemented *elo-3* RNAi animals with the Sph extract and monitored the survival under glucose stress. Here the Sph was able to partially rescue the shortened lifespan of *elo-3* RNAi animals under glucose stress from 6.0 ± 1.4 days to 7.6 ± 2.0 days (Fig. 5D). Interestingly, the Sph supplement extended the mean lifespan of control elo*-3* animals from 12.5 ± 2.1 days to 14.3 ± 1.9 days suggesting that the *elo-3* RNAi background enhances the impact of Sph supplementation (Fig. 5D).

**Fig. 6:**
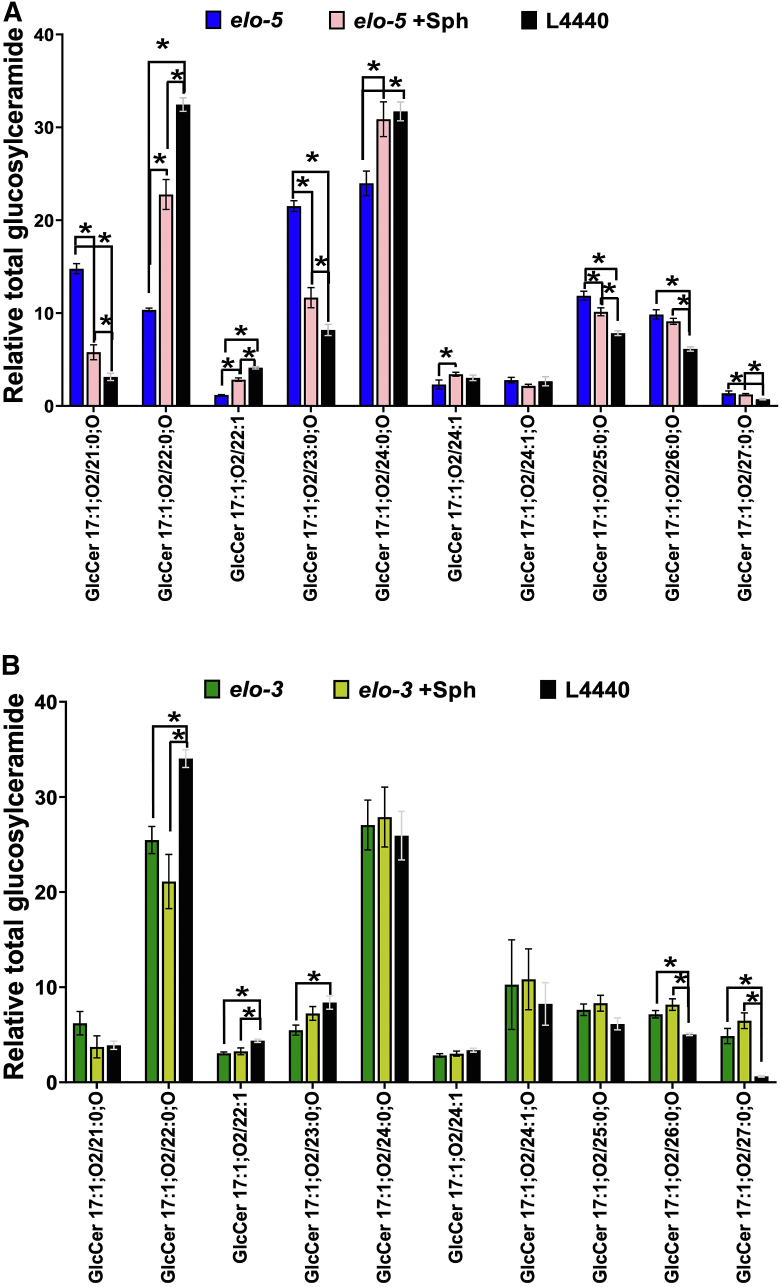
Glucosylceramide profile of *elo-5* and *elo-3* animals supplemented with sphingolipids. A: Synchronized L1 animals were treated with L4440 or *elo-5* RNAi for 48 h and then transferred to NGM +CI plates supplemented with 500 μl sphingolipid (Sph) extract or ethanol control for 18 h. The sphingolipids were extracted and analyzed, and the results show a significant increase of 22;0;O GlcCer in *elo-5* +Sph compared with *elo-5* from 10.34 ± 0.19% to 22.77 ±1.61%. In addition, there were other significant changes in multiple species and some species were stable. B: Similarly, the glucosylceramide profile of *elo-3* animals supplemented with Sph extract was analyzed. Surprisingly, there was no significant difference between *elo-3* controls and *elo-3* animals supplemented with Sph extract (see supplemental Table S7 for full list). Data were generated from three independent biological replicates. Statistical significance was calculated by multiple *t*-test corrected by false discovery rate, with an adjusted p-value (q) at 5%.

It is possible that the Sph supplementation rescues the animal’s ability to survive on glucose stress because the Sph pool contains mmBCFA. One of the challenges of metabolic studies is that we cannot discount the possibility that the mmBCFA supplementation and not intact GlcCer supplementation is responsible for the rescue. In fact, lipid absorption in the gut would necessitate GlcCer is broken down before absorption can occur. To understand more about the supplementation, we analyzed the GlcCer pool from the Sph-supplemented animals by HPLC-MS/MS. There are no significant changes in the GlcCer with Sph supplementation in control nematodes; however, there are many changes in the *elo-5* RNAi animals fed the supplement (Fig. 6A). Most importantly, there is a significant increase in 22:0;O GlcCer from 10.35 ± 0.19% to 22.77 ± 1.61% in *elo-5* RNAi upon supplementation. There are also significant increases in 22:1 GlcCer (1.19 ± 0.04% to 2.84 ± 0.18%), 24:0;O GlcCer (23.97 ± 1.33% to 30.87 ± 1.87%), and 24:1 GlcCer (2.32 ± 0.47% to 3.42 ± 0.21%). Importantly, there are also GlcCer species that are decreased significantly upon supplementation including 21:0;O GlcCer (14.78 ± 0.55% to 5.78 ± 0.80%), 23:0;O GlcCer (21.51 ± 0.58% to 11.66 ± 1.09%), and 25:0;O GlcCer (11.86 ± 0.48% to 10.14 ± 0.42%). Again, there is distinct regulation of odd-chain versus even-chain GlcCers. The remaining GlcCer species were stable regardless of supplementation (see supplemental Table S6 for full list). Although we cannot determine if there is an increase in GlcCer production or absorption, it is clear that the GlcCer pool is much more similar to WT Sph levels following supplementation (Fig. 6A). Even though the rescue was less pronounced in *elo-3* RNAi-fed animals, we profiled the GlcCer in those animals as well (Fig. 6B). In doing so, we found no significant changes with WT Sph exposure; however, it is important to note that the *elo-3* animals have a much more similar GlcCer profile to WT animals to begin with (see supplemental Table S7 for full list). We believe that ELO-5 plays a more prominent role in regulating the GlcCer pool upon glucose exposure. Taken together with the small-scale recovery from glucose sensitivity, the lipidomic profiling supports the conclusion that the mmBCFA synthesis via ELO-5 is critical to produce certain GlcCer species, specifically 22:0;O GlcCer.

## Discussion

In this study, we have established 22:0;O GlcCer as a critical mediator of the response to glucose in *C. elegans.* The monomethyl branched-chain fatty acids and this derived GlcCer has been previously implicated in postembryonic development and foraging in *C. elegans* (18, 19). Here, we define a new role for this Sph in participating in the nematode’s response to high-glucose diets. In doing so, ELO-5, ELO-3, and CGT-3, all enzymes involved in 22:0;O GlcCer production, have been established as participants in the system of metabolic pathways that are critical for surviving high-glucose diets. The response network to glucose stress in *C. elegans* has been developing over the last decade, and our work here expands the knowledge of how this response acts mechanistically.

One of the striking features of these glucose-response targeted lipidomic datasets is the lack of significant changes in wild-type animals. In fact, we find no significant changes in animals fed 100 mM glucose in our initial global comparison, highlighting the rewiring of metabolic pathways with glucose. This maintenance of overall phospholipid composition is somewhat unexpected as a few changes in fatty acid composition have been documented (9, 51). We hypothesize that the existence of so many individual phospholipid species dilutes the impact of the fatty acid changes on any given lipid. The adjustment in metabolic pathways can be observed by compromising the genes in pathways required for the response including the membrane sensor, PAQR-2. PAQR-2 has been established as a critical mediator of the transcriptional changes that occur with glucose exposure including increased expression of desaturases and elongases required to produce unsaturated fatty acids and monomethyl branched-chain fatty acids (6, 9). Here, we have found that the role of mmBCFAs in this response is in providing the precursors for GlcCer synthesis; thus, the PAQR-2 sensor may be connected to Sph metabolism. This is of particular interest as GlcCer is a mediator of TOR activity in other conditions (19), although further studies would be needed to confirm the role of TOR in glucose response.

This study revealed interesting membrane remodeling seen in *elo-5* RNAi-fed animals under glucose. In general, the results show that *elo-5*-fed RNAi worms under glucose stress had relatively fewer double bonds and an overall shorter chain length. This shift is characteristic of an adaptation of the membrane to a phospholipid composition that is resistant to oxidative stress as highly polyunsaturated fatty acids are most susceptible to damage by reactive oxygen species (52, 53). We hypothesize the GlcCer production helps promote a response to the high level of glucose, and therefore, the absence of ELO-5 would increase the oxidative burden in the cell. The higher oxidative load would then promote a shift in membrane composition that would be protective against the higher levels of oxidative species seen in high-glucose diets (54, 55). Although it has been hypothesized that glucose supplementation results in excess saturated fatty acid in the membrane (6, 8), we believe that the extra glucose may be increasing the oxidative load in the animal, and this shift lends support to that model.

RNAi of CGT led to a perturbation in GlcCer populations and a dramatically shortened lifespan on glucose. We were intrigued by the fact that *cgt-3* RNAi needed a longer timeframe to see significant decreases in Sph populations while compromised survival happens readily. In fact, the longer RNAi period required for the lipidomic analysis results in a significant death of the treated animals with only 80 ± 3.1% animals remaining at time of collection. The rapid rate of death in *cgt-3* RNAi suggests an additional role for CGT-3 that may be independent of the Sph regulation. We believe that this rapid death may be explained by the accumulation of a toxic intermediate, which will be the subject of future studies. In addition, it is possible that the role of GlcCer in glucose response may be required only in a specific subset of cells within the intestine. In support of this tissue specificity, studies in the nematode have found that mutations in any of the three CGT genes can be rescued by expression in a small subset of intestinal cells (29). Many lipid signaling pathways have been found to work in a cell nonautonomous manner, and it is likely that the GlcCer response to glucose follows that pattern.

One of the major challenges of metabolic studies is proving causality between a single lipid species and the phenotype of interest. The interconnected nature of metabolic pathways makes it impossible to cleanly test how that species of interest works as compromising its levels and rescue studies will impact other intermediates of the pathway and have a ripple effect on the metabolism of the animals. For example, here we identify 22:0;O GlcCer as a potential specific regulator of glucose response and we can compromise the production of that species with *elo-5* RNAi. However, in doing so, we also perturb many additional Sph pools. Other genes within the synthesis pathway can be used to corroborate the role of 22:0;O GlcCer, but it is also important to note that perturbations at different enzymes leads to unique Sph profiles. Finally, 22:0;O GlcCer is not commercially available, but we can supplement WT Sph to look for rescue. The process of lipid absorption where lipase activity removes the fatty acid tails before absorption into cells may impact the Sph pool. Although the options for direct testing are limited, we believe that the results presented here viewed in total provide a strong argument that 22:0;O GlcCer is largely responsible for the glucose response.

Our study here highlights the power of mass spectrometry in the genetic organism *C. elegans.* Stable isotope labeling originally identified mmBCFA as implicated in the response to glucose. In fact, this role was only observed using stable isotopes as the populations of these fatty acids are not significantly altered in wild-type nematodes (9). The stability of lipid populations is a common trend in wild-type worms; however, by combining stable isotope and genetic approaches, it is clear that multiple pathways are required to maintain the membrane populations under these stressed conditions. In fact, reduction or elimination of these pathways including ELO-5 and CGT-3 led to reduced survival under high-glucose diets. Overall, the utilization of mass spectrometry methods combined with genetic tools has identified a novel and specific role for a particular GlcCer in membrane adaptation.

## Data availability

The raw lipidomics data from HPLC/MS-MS measurements are available upon request.

## Supplemental data

This article contains supplemental data.

## Conflict of interest

The authors declare that they have no conflicts of interest with the contents of this article.
