# Supplementary material for: Targeted lipidomics reveals a novel role for glucosylceramides in glucose response
Source: J Lipid Res. 2023 May 26;64(7):100394. doi: 10.1016/j.jlr.2023.100394 (PMC10320606; doi:10.1016/j.jlr.2023.100394)
Supplement: Supplemental Figure S1 [file mmc8.docx]

**SUPPLEMENTAL INFORMATION:**

# Targeted Lipidomics Reveals a Novel Role for Glucosylceramides in Glucose Response

**Mark A. Xatse^1^, Andre F. C. Vieira^1^, Chloe Byrne ^1^, and Carissa Perez Olsen^1,#^**

^1^From the Department of Chemistry and Biochemistry, Worcester Polytechnic Institute, Worcester, Massachusetts, USA


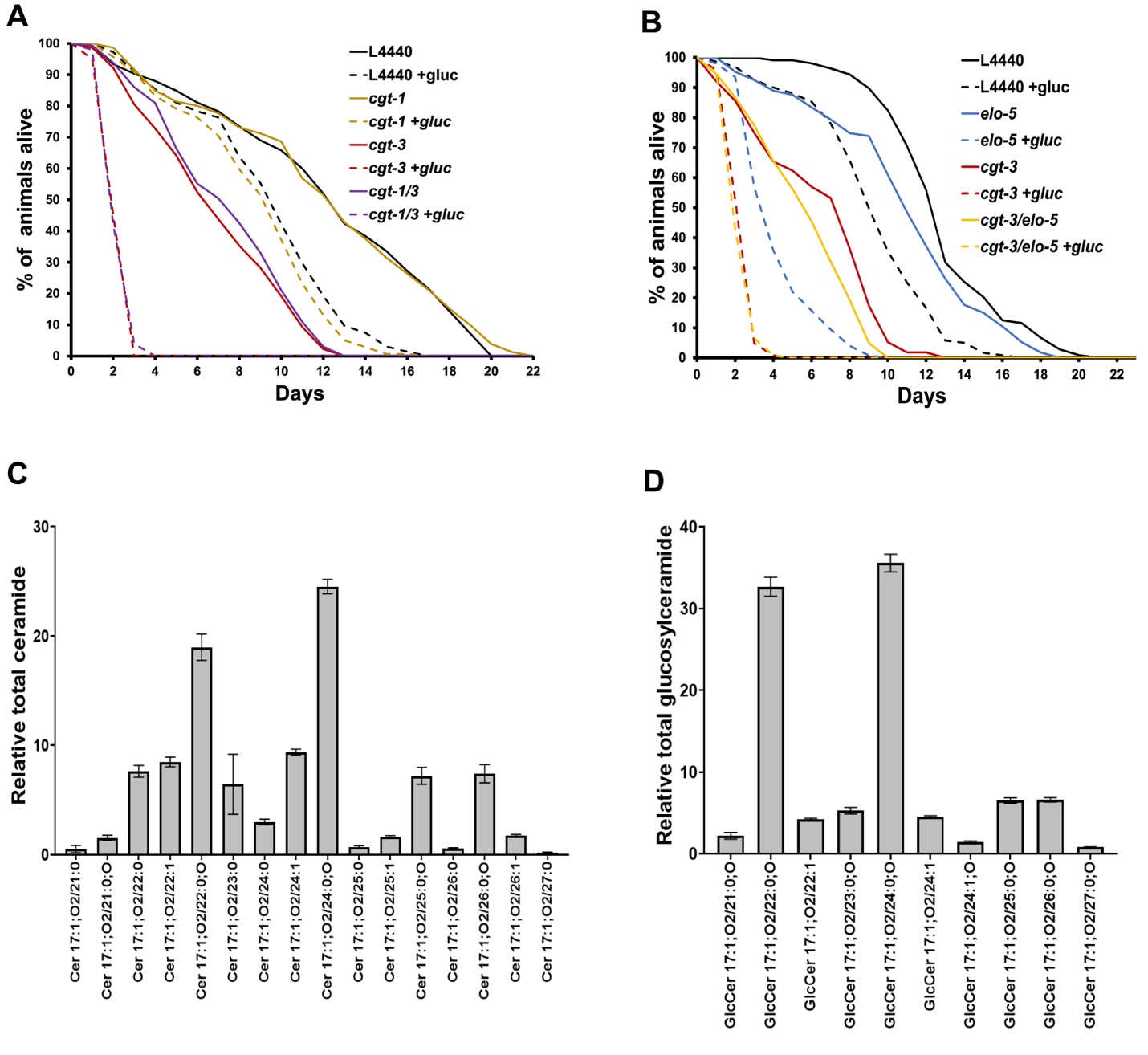


**Figure S1: CGT-1 does not decrease the survival of animals fed with 100 mM glucose**.

**A)** RNAi knockdown of *cgt-1* (dark yellow) and control (black) in N2 worms was initiated from the L1 stage. After 48 hours (L4 stage), the animals were transferred to NGM+CI plates with (dashed line) or without (solid line) 100 mM glucose (+gluc). There was no significant difference between *cgt-1* + gluc (yellow dashed) glucose compared to L4440 +gluc (black dashed). The combination of *cgt-3* and *cgt-1* RNAi (1:1) did not further decrease the shortened lifespan under glucose stress. **B)** Combination of *cgt-3* and *elo-5* at 1:1 ratio (yellow) did not further exacerbate the shortened lifespan of *cgt-3* RNAi under glucose stress. Sphingolipid profile of extract for supplementation. Briefly synchronized L1 wild type animals were grown for 66 hours. Sphingolipid were extracted and purified as shown in the methods and resuspended at a density of ~ 50,000 animals in 5 ml of ethanol. 500 µL of extract was dried down and resuspended in 200 µL isopropanol/chloroform/methanol (90:5:5, v/v/v) for HPLC-MS/MS analysis. The bar chart shows the **C)** ceramide where 22:0;O Cer and 24:0;O Cer constitute 18 % and 24 %, respectively. **D)** Glucosylceramide profile of the extract where the 22:0;O GlcCer and 24:0;O Cer constitute 32 % and 35 %, respectively.
